# The mitochondrial genomes of sarcoptiform mites: are any transfer RNA genes really lost?

**DOI:** 10.1186/s12864-018-4868-6

**Published:** 2018-06-18

**Authors:** Xiao-Feng Xue, Wei Deng, Shao-Xuan Qu, Xiao-Yue Hong, Renfu Shao

**Affiliations:** 1 Department of Entomology, Nanjing Agricultural University, Nanjing, 210095 Jiangsu China; 2 Institute of Vegetable Crops, Jiangsu Academy of Agricultural Sciences, Nanjing, 210014 Jiangsu China; 3 GeneCology Research Centre, Centre for Animal Health Innovation, School of Science and Engineering, University of the Sunshine Coast, Maroochydore, QLD 4556 Australia

**Keywords:** Mitochondrial genome, tRNA structure, *Histiostoma feroniarum*, *Rhizoglyphus robini*

## Abstract

**Background:**

Mitochondrial (mt) genomes of animals typically contain 37 genes for 13 proteins, two ribosomal RNA (rRNA) genes and 22 transfer RNA (tRNA) genes. In sarcoptiform mites, the entire set of mt tRNA genes is present in *Aleuroglyphus ovatus*, *Caloglyphus berlesei*, *Dermatophagoides farinae*, *D. pteronyssinus*, *Histiostoma blomquisti* and *Psoroptes cuniculi*. Loss of 16 mt tRNA genes, however, was reported in *Steganacarus magnus*; loss of 2–3 tRNA genes was reported in *Tyrophagus longior*, *T. putrescentiae* and *Sarcoptes scabiei*. Nevertheless, convincing evidence for mt gene loss is lacking in these mites.

**Results:**

We sequenced the mitochondrial genomes of two sarcoptiform mites, *Histiostoma feroniarum* (13,896 bp) and *Rhizoglyphus robini* (14,244 bp). Using tRNAScan and ARWEN programs, we identified 16 and 17 tRNA genes in the mt genomes of *H. feroniarum* and *R. robini*, respectively. The other six mt tRNA genes in *H. feroniarum* and five mt tRNA genes in *R. robini* can only be identified manually by sequence comparison when alternative anticodons are considered. We applied this manual approach to other mites that were reported previously to have lost mt tRNA genes. We were able to identify all of the 16 mt tRNA genes that were reported as lost in *St. magnus*, two of the three mt tRNA genes that were reported as lost in *T. longior* and *T. putrescentiae*, and the two mt tRNA genes that were reported as lost in *Sa. scabiei*. All of the tRNA genes inferred from these manually identified genes have truncation in the arms and mismatches in the stems.

**Conclusions:**

Our results reveal very unconventional tRNA structures in sarcoptiform mites and do not support the loss of mt tRNA genes in these mites. The functional implication of the drastic structural changes in these tRNA genes remains to be investigated.

**Electronic supplementary material:**

The online version of this article (10.1186/s12864-018-4868-6) contains supplementary material, which is available to authorized users.

## Background

Mitochondria are critical organelles for cellular energy production in eukaryotes. The four protein complexes in the respiratory chain are encoded by both mitochondrial (mt) genomes and nuclear genomes [1]. For animals, mt genomes typically have 37 genes for 13 proteins, two ribosomal RNA (rRNA) genes and 22 transfer RNA (tRNA) genes [2]. The proteins are essential for the formation of respiratory chain, and the rRNA genes and tRNA genes are essential for the translation process. Loss of genes is rare in animal mt genomes, but has been reported or suggested in several lineages, e.g., loss of *atp8* in nematodes [3], bivalves [4], cnidarians [5] and flatworms [6], loss of *trnD* in scorpions [7], and loss of both protein-coding and tRNA genes in mites [8, 9].

Mites and ticks (subclass Acari) represent a major group (> 54,000 species) in the class Arachnida [10] and colonize a wide range of terrestrial, marine and aquatic habitats [11]. The order Sarcoptiformes comprises approximately 16,300 extant species [12]. Some species are medically important, e.g. house dust mites causing allergic symptoms in humans [13, 14], scabies mite infecting skin of humans and other animal species [15]. Some species are also economically important, e.g. acarid mites inhabiting stored food products [16]. Mt. genomes of 40 species of mites and 48 species of ticks have been sequenced (as of records from database of NCBI on 1 August 2017). Loss of mt tRNA genes has been reported in four sarcoptiform mites from three superfamilies: 16 in *Steganacarus magnus* (Phthiracaroidea) [8], three (*trnF*, *trnS*_*1*_ and *trnQ*) in two *Tyrophagus* species (Acaroidea) [17, 18], and two (*trnA* and *trnY*) in *Sarcoptes scabiei* (Sarcoptoidea) [15]. Edwards et al. reanalyzed the mt genome sequence of *St. magnus* and identified three of the 16 tRNA genes reported as lost [19]. On the other hand, the full set of tRNA genes were found in six other species of sarcoptiform mites from four superfamilies: *Aleuroglyphus ovatus* [20], *Caloglyphus berlesei* [21], *Dermatophagoides farinae* [22], *D. pteronyssinus* [23], *Histiostoma blomquisti* [24] and *Psoroptes cuniculi* [25]. Thus, the evidence available is conflicting whether or not any tRNA genes are really lost in sarcoptiform mites. If tRNA genes were indeed lost, to what extent did it occur? If no tRNA genes were lost, why couldn’t they be identified in some sarcoptiform mites? To address these questions and to lay a solid foundation for comparative studies of the mt genomes of sarcoptiform mites, we sequenced the mt genomes of two more species of sarcoptiform mites, *Rhizoglyphus robini* and *Histiostoma feroniarum*, from the superfamilies Acaroidea and Histiostomatoidea respectively, and compared the mt genome sequences of all of the sarcoptiform mites available to date.

## Results

### Mitochondrial genomes of *Rhizoglyphus robini* and *Histiostoma feroniarum*

**Fig. 1:**
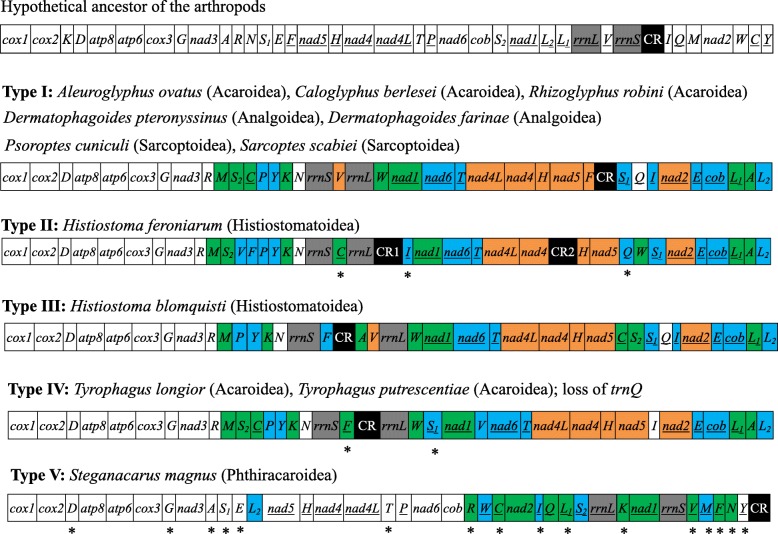
Mitochondrial gene arrangements in the sarcoptiform mites. Underlined genes are on the N-strand. Translocated or inverted genes are color-coded (blue: inversion and translocation; green: translocation; orange: inversion). rRNA genes are in grey; control regions are in black. Abbreviations of protein-coding genes are: *atp6* and *atp8* for ATP synthase subunits 6 and 8; *cox1–3* for cytochrome oxidase subunits 1–3; *cob* for cytochrome b; *nad1–6* and *nad4L* for NADH dehydrogenase subunits 1–6 and 4 L; *rrnL* and *rrnS* for large and small rRNA subunits; tRNA genes are indicated by the single-letter IUPAC-IUB abbreviations for their corresponding amino acids. Type I: a common pattern among sarcoptiform mites, found in different genera and families. Type II: same as Type I except for the translocation of three tRNA genes (*trnV*, *trnW* and *trnF*). Type III: same as Type I except for the translocation of four tRNA genes (*trnS*_*2*_, *trnC*, *trnF* and *trnA*). Type IV: similar to Type I but *trnV* was translocated and *trnI* was reversed except for the loss of three tRNA genes. Type V: very different from Type I. Asterisks under tRNA genes indicate those retrieved by our manual approach

The mt genomes of *R. robini* and *H. feroniarum* are 14,244 bp and 13,896 bp long, respectively. Like other mites and ticks reported previously, the mt genomes of *R. robini* and *H. feroniarum* are circular and have the 13 protein-coding genes (PCGs) and two rRNA genes (Fig. 1, Additional file 1: Figure S1, Additional file 2: Table S1 and Additional file 3: Table S2). Using tRNAscan-SE [26] and ARWEN [27] programs, we identified 16 and 17 tRNA genes in the mt genomes of *H. feroniarum* and *R. robini*. The other six mt tRNA genes of *H. feroniarum* (*trnR*, *trnM*, *trnS*_*2*_, *trnY*, *trnS*_*1*_, *trnA*) and the other five mt tRNA genes of *R. robini* (*trnR*, *trnM*, *trnY*, *trnS*_*1*_, *trnA*) could only be identified manually by sequence alignment and secondary structure comparison with those identified in other species of sarcoptiform mites [8, 15, 18, 20–25, 28]. The putative mt tRNA genes were highly truncated in both *H. feroniarum* (48 to 61 bp) and *R. robini* (47 to 63 bp) (Additional file 1: Figure S1), missing either D-arm or T-arm, except tRNA-Lys, which has the typical cloverleaf secondary structure in both mites. Further, the putative tRNA-Arg of *R. robini* does not have a D-arm, nor a T-arm. Most of the putative tRNA genes also have mismatches on T-arm, D-arm, acceptor arm or anticodon arm (Additional file 1: Figure S1).

Genes are on both strands of the mt genomes of *R. robini* and *H. feroniarum*. In *R. robini*, J-strand has 26 genes whereas the N-strand has 11 genes. In *H. feroniarum*, J-strand has 27 genes whereas the N-strand has 10 genes. The start codons of the 13 PCGs were ATN and the stop codons were TAA or TAG in both *H. feroniarum* (Additional file 3: Table S2) and *R. robini* (Additional file 2: Table S1). Incomplete stop codons, T, was found in protein-coding genes that precede a tRNA gene in *R. robini*. The two longest non-coding regions (NCRs) in *H. feroniarum* are 100 bp in size between *rrnL* and *trnI* and 143 bp between *nad4* and *trnH* (Fig. 1). We annotated these two NCRs as the putative control regions (CR) of *H. feroniarum*. The putative CR of *R. robini* is 319 bp between *trnF* and *trnS*_*1*_. No conserved sequences were found between the CRs of the two sarcoptiform mites. No other NCRs longer than 100 bp were found in the mt genomes of these two mites.

Most of the mt genes are rearranged in *R. robini* and *H. feroniarum* relative to the inferred ancestral mt genome of arthropods (Fig. 1) [2, 29]. Currently five types of mt gene order were found in the 12 species the sarcoptiform mites, for which mt genomes have been sequenced (Fig. 1). *R. robini* has a common Type I gene order, which has been found in seven other species from different genera and families. *H. feroniarum* has a Type II gene order, which is only found in this species. Type III and Type V gene orders are also restricted to single species. Two *Tyrophagus* species have Type IV gene order (Fig. 1).

### Retrieving the “lost” mt tRNA genes in sarcoptiform mites

**Fig. 2:**
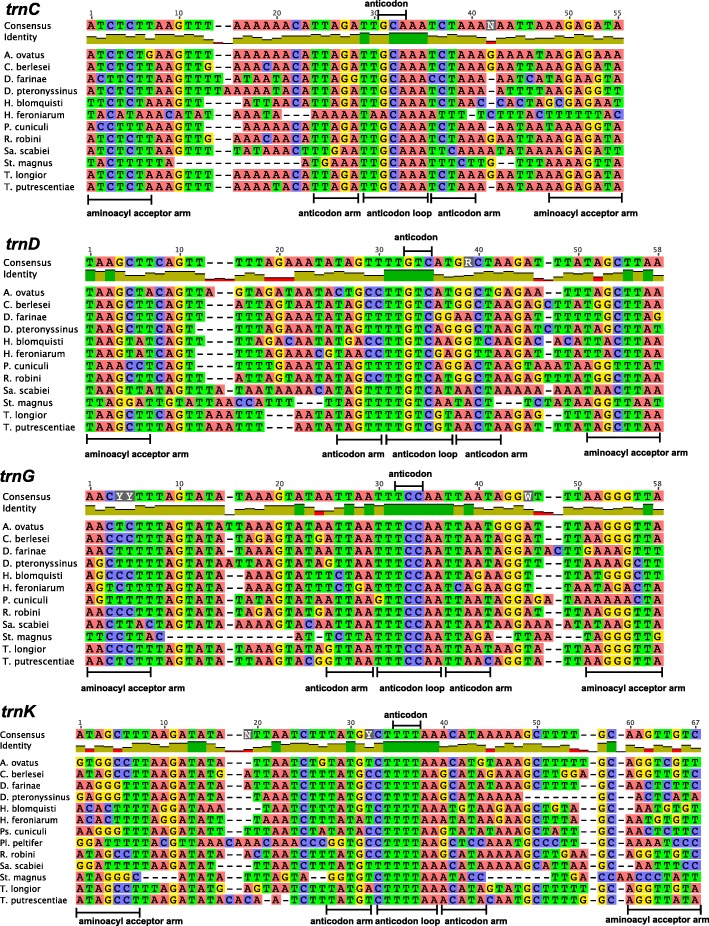
The alignment of nucleotide sequences of 4 mitochondrial tRNA genes (*trnC*, *trnD*, *trnG and trnK*) of sarcoptiform mites. The conserved sequences of aminoacyl acceptor arm, anticodon arm, anticodon loop and anticodon were marked

**Fig. 3:**
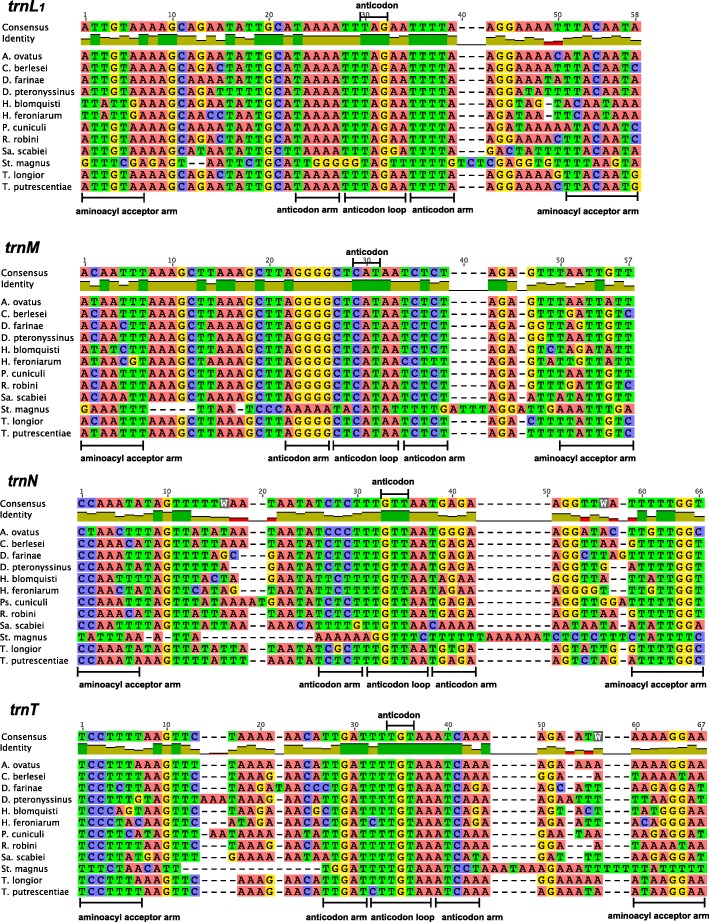
The alignment of nucleotide sequences of 4 mitochondrial tRNA genes (*trnL*_*1*_, *trnM*, *trnN* and *trnT*) of sarcoptiform mites. The conserved sequences of aminoacyl acceptor arm, anticodon arm, anticodon loop and anticodon were marked

**Fig. 4:**
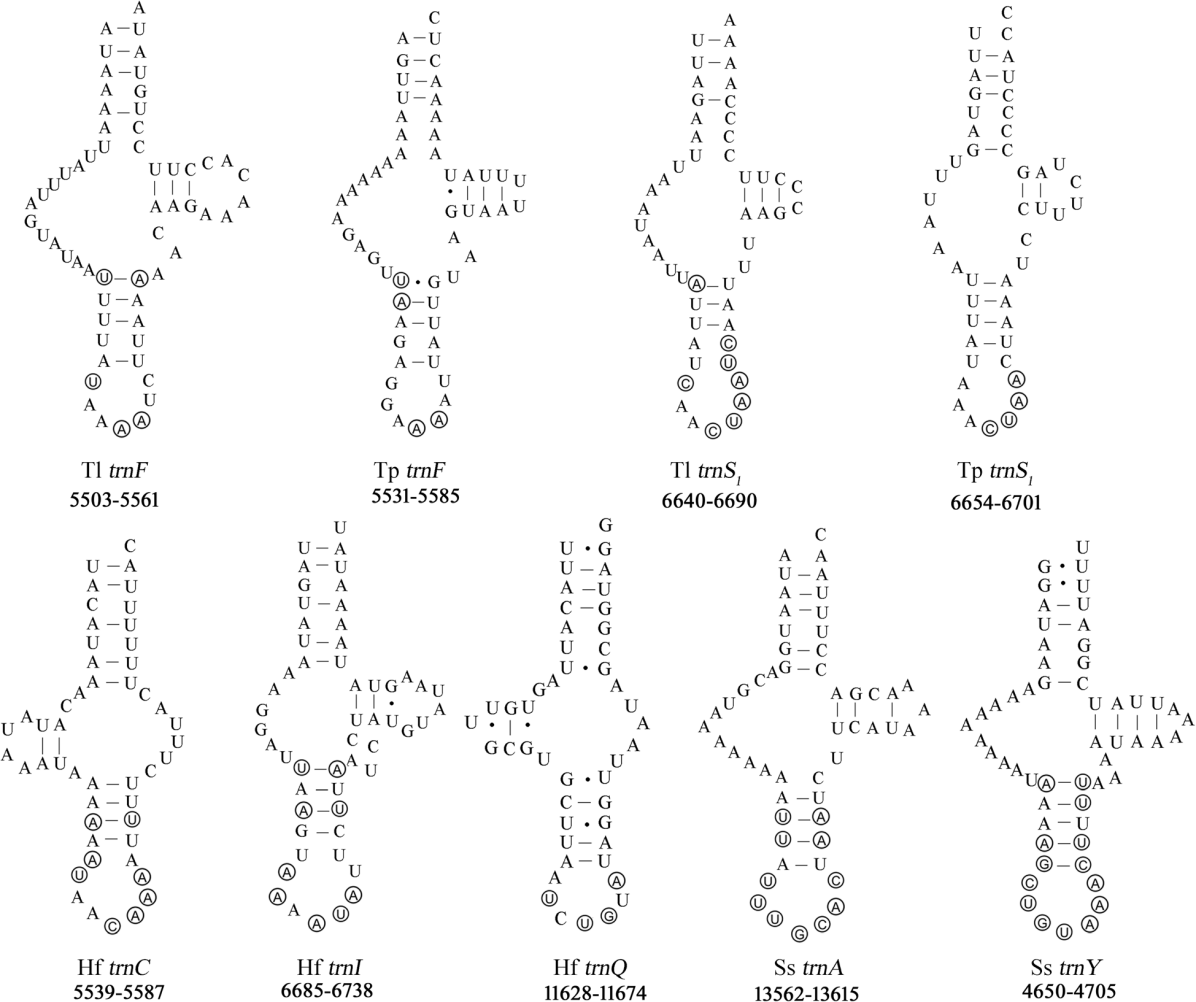
The secondary structure of three putative mitochondrial tRNA genes of *Histiostoma feroniarum* (Hf), two tRNA genes of *Tyrophagus longior* (Tl), two tRNA genes of *Tyrophagus putrescentiae* (Tp) and two tRNA genes of *Sarcoptes scabiei* (Sc) retrieved by our manual approach. tRNA genes are labeled with the abbreviations of their corresponding amino acids. Dashes indicate Watson–Crick bonds; dots indicate bonds between U and G. Shared identical sequences (Fig. 2 and Additional file 4: Figure S2) of anticodon arm and anticodon loop among sarcoptiform mites are circled. The position of each tRNA is indicated below its secondary structure

**Fig. 5:**
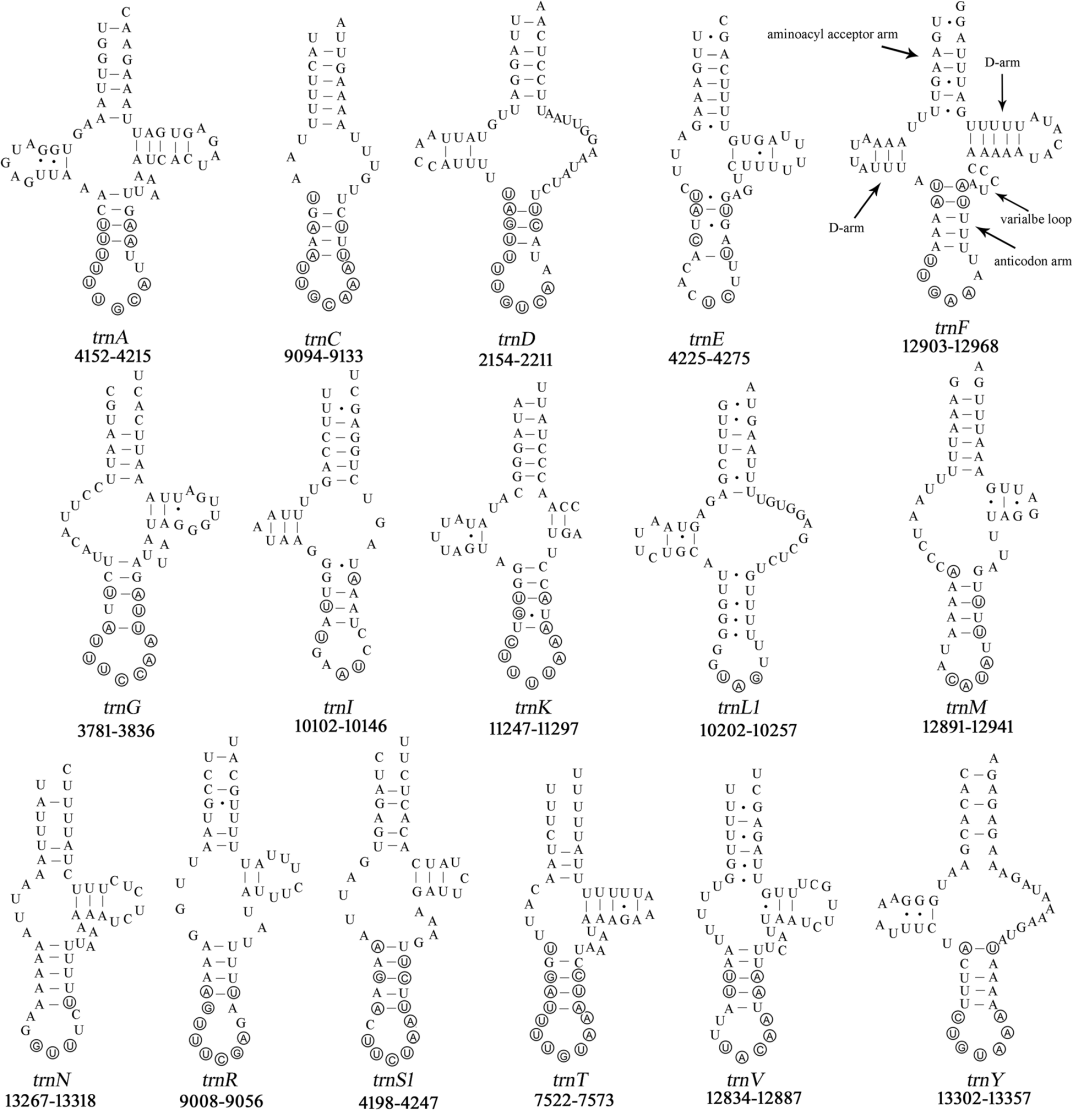
The secondary structure of the 16 putative mitochondrial tRNA genes of *Steganacarus magnus* retrieved by our manual approach. tRNA genes are labeled with the abbreviations of their corresponding amino acids. Dashes indicate Watson–Crick bonds; dots indicate bonds between U and G. Shared identical sequences (Figs. 2, 3 and Additional file 4: Figure S2) in anticodon arm and anticodon loop among sarcoptiform mites are circled. The position of each tRNA is indicated below its secondary structure

We applied the manual tRNA gene search approach above to *St. magnus*, *T. longior*, *T. putrescentiae* and *Sa. scabiei*, which were reported to have lost 2 to 16 tRNA genes in their mt genomes [8, 15, 17, 18]. These “lost” tRNA genes cannot be identified by tRNAscan-SE nor ARWEN programs with all possible parameters we tested. In our manual approach, we focused on the gap regions (46–215 bp) between identified genes where the anticodon sequences of the “lost” tRNA genes can be identified. We then aligned the sequences of these gap regions with those of the candidate tRNA genes identified in other species of sarcoptiform mites, and compared the inferred secondary structure with each other (Fig. 2, 3 and Additional file 4: Figure S2). Based on the overall secondary structure, we retrieved the two “lost” tRNA genes, *trnA* and *trnY*, in *Sa. scabiei* (Fig. 4) and all of the 16 “lost” tRNA genes (*trnC*, *trnG*, *trnK*, *trnT*, *trnY*, *trnA*, *trnD*, *trnR*, *trnS*_*1*_, *trnF*, *trnV*, *trnL*_*1*_, *trnM*, *trnN*, *trnE* and *trnI*) in *St. magnus* (Fig. 5). The two “lost” tRNA genes, *trnF* and *trnS*_*1*_, were also retrieved in *T. longior* and *T. putrescentiae* (Fig. 4), whereas *trnQ* was not retrieved by our manual approach. Of the 16 tRNA genes of *St. magnus* retrieved manually, five tRNA genes (*trnC*, *trnD*, *trnG*, *trnK* and *trnT*) are more conserved in nucleotide sequence than the other 11 tRNA genes (*trnA*, *trnE*, *trnF*, *trnI*, *trnL*_*1*_, *trnM*, *trnN*, *trnR*, *trnS*_*1*_, *trnV* and *trnY*) when compared with those of other species of sarcoptiform mites (Figs. 2, 3 and 5)*.* The secondary structure inferred from these manually retrieved tRNA gene sequences is either T-armless or D-armless, and has 1–2 mismatches on the AC-stem or 1–4 mismatches on AA-stem (Figs. 4 and 5). Apparently, the severe truncation and mismatches contribute to the failure of identifying these tRNA genes by tRNAscan-SE and ARWEN programs. Furthermore, less common anticodon sequences are seen in a number of tRNA genes that we identified manually: *trnE* (CUC instead of UUC) and *trnI* (AAU instead of GAU) of *St. magnus* (Fig. 5); *trnC* (ACA instead of GCA), *trnI* (AAU instead of GAU) and *trnQ* (CUG instead of UUG) of *H. feroniarum* (Fig. 4); *trnF* (AAA instead of GAA) and *trnS*_*1*_ (ACU instead of GCU) of *T. longior* and *T. putrescentiae* (Fig. 4).

It is noteworthy that four “lost” tRNA genes of *St. magnus*, *trnE*, *trnG*, *trnP* and *trnS*_*1*_, were also retrieved by Edwards et al. [19]; two of these tRNA genes, *trnG* and *trnS*_*1*_, were at the same location as we inferred, but were different in the sequences of AA-stems. *trnE* was abnormally inferred within *nad5* by Edwards et al. [19], whereas we retrieved *trnE* in a gap where no other genes were found. Clearly, the comparative approach we used has its limits. Further evidence is required to establish the exact locations of the “lost” tRNA genes of *St. magnus*, such as transcriptional data of its mt tRNA, and sequence comparison among closely related *Steganacarus* species.

### Codon usage in the mt genomes of sarcoptiform mites

**Fig. 6:**
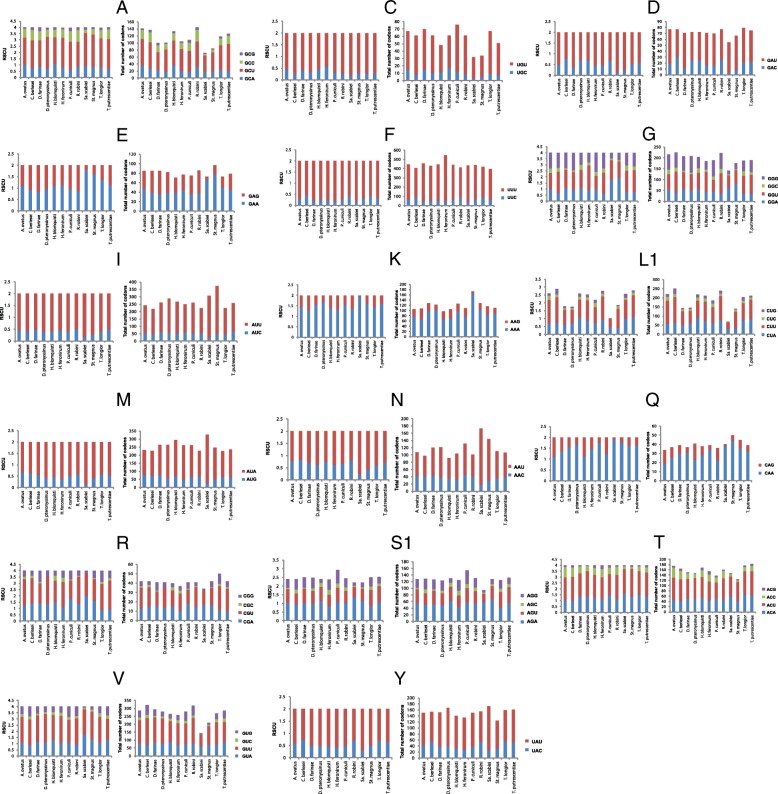
Relative synonymous codon usage (RSCU) and codon numbers of 17 amino acids (**a**, **c**, **d**, **e**, **f**, **g**, **i**, **k**, **l**_**1**_, **m**, **n**, **q**, **r**, **s**_**1**_, **t**, **v** and **y**) in the mitochondrial (mt) genomes of sarcoptiform mites. The X-axis indicates the sarcoptiform mites; the Y-axis indicates the RSCU or total number of codons. The blue column indicates the codons that are complimentary to the anticodons of their corresponding mt tRNA. The red, green and purple columns indicate the imperfect, synonymous codons to the anticodons of their corresponding mt tRNA genes

There is no evidence yet that nuclear tRNA genes can be imported into mitochondria in mites or other animals. If a mt tRNA gene is indeed lost and there is no nuclear replacement, then its corresponding codons in the mt protein-coding genes will not be translated. We analyzed the codon usage in the mt protein-coding genes of the 12 species of sarcoptiform mites to see whether or not, and how, the corresponding codons of the “lost” tRNA genes are used. Overall, the codon use is very similar across all sarcoptiform mites. The most frequently used codons are for amino acids Phe (11.2–15.3%), Leu (7.1–10.0%), Met (6.3–10.0%), Ser (7.4–9.5%), Val (4.4–8.8%) and Ile (6.1–10.6%) (Additional file 5: Table S3, Fig. 6, Additional file 6: Figure S3). The least used codons are for Gln (0.9–1.4%), Cys (1.0–2.1%), Arg (1.0–1.4%), His (1.5–1.8%) and Trp (1.9–2.8%) (Additional file 5: Table S3). Codons for all of the 22 amino acids are present in all of the protein-coding genes in all of the sarcoptiform mites, including the four species in which mt tRNA gene “loss” has been reported (Fig. 6). The frequency of each codon is also very similar across all of the sarcoptiform mites.

## Discussion

Mitochondria have their own transcription and translation systems, separate from the nuclear systems [2]. The tRNA genes encoded by mt genomes are critical to the mt translation system. With few exceptions, animal mt genomes encode 22 tRNA genes for the 20 amino acids used in protein synthesis [30]. Loss of any of the 22 mt tRNA genes will severely affect the translation system in mitochondria unless a nuclear equivalent can be imported into mitochondria. Loss of tRNA genes have been reported in the mt genomes of four species of sarcoptiform mites [8, 15, 17, 18]. However, convincing evidence for the loss of mt tRNA genes in these mites is lacking; alternative possibilities have not been explored. Here, we show that all of the “lost” tRNA genes except one (*trnQ* of *T. putrescentiae*) can be found by a manual comparative approach [31]. Furthermore, our codon use analysis does not support the loss of any of the 22 mt tRNA genes in sarcoptiform mites because the overall codon usage is very similar across all sarcoptiform mites including those in which mt tRNA genes were reported as lost [8, 15, 17, 18].

Instead of loss, we propose that it is the highly unusual secondary structure of inferred mt tRNA genes that makes them unidentifiable by the tRNA search programs. The mt tRNA genes of animals usually possess a cloverleaf secondary structure with four arms: AA-arm, D-arm, AC-arm and T-arm [32]. The only exception is the tRNA for serine (anticodon GCT), which lack a D-arm in nearly all animals; this is apparently an ancestral feature for animals [32]. Using a manual comparative approach, we were able to identify all of the 16 “lost” tRNA genes in *St. magnus*, three in *H. feroniarum*, two of the three in *T. longior* and *T. putrescentiae*, and two in *Sa. scabiei*. These retrieved tRNA genes are either D-armless or T-armless and have many mismatches at AA-stems and AC-stems. Clearly, the highly unconventional structure prevented them from being found by the tRNA search programs.

Post-transcriptional tRNA editing is likely common and necessary in the mitochondria of sarcoptiform mites. Mt. tRNA editing has been reported in centipede [33], velvet worms [34] and sponges [35], where nucleotides can be substituted, inserted and/or deleted from transcripts. Mt. tRNA editing has largely been found at the 3′ end of the AA-stems in metazoan [33–36], also in rebuilding T-arm, variable loop or even AC-stem [34]. Within the class Arachnida, mt tRNA editing was suggested to form AA-stems in spiders [37, 38]. Investigation into tRNA editing in sarcoptiform mites is apparently needed.

## Conclusions

In summary, using a manual comparative approach, we were able to identify all of the mt tRNA genes which were reported previously as lost in sarcoptiform mites except for *trnQ* of *T. putrescentiae*. Our codon usage analysis does not support the loss of any mt tRNA genes in sarcoptiform mites. Instead, the mt tRNA genes reported as “lost” previously in sarcoptiform mites have unusual secondary structures and contain many nucleotide mismatches. Post transcriptional tRNA editing is likely common and necessary in sarcoptiform mites and need to be investigated in future studies.

## Methods

### Collection of mites

*H. feroniarum* and *R. robini* were collected from mushrooms (*Pleurotus ostreatus*) in March 2015 at the Institute of Vegetable Crops, Jiangsu Academy of Agricultural Sciences, Nanjing, China. Mite samples were either used immediately for DNA extraction or were preserved in 100% ethanol at − 20 °C prior to DNA extraction. Samples of each mite species were also mounted to slides as voucher, using Hoyer’s medium for morphological check with Zeiss A2 (microphoto camera AxioCam MRc) microscope. All of the specimens and vouchers were deposited at the Arthropod Collection, Department of Entomology, Nanjing Agricultural University, China.

### DNA extraction, mt genome amplification and sequencing

Genomic DNA was extracted from individuals, using a DNeasy Blood and Tissue Kit (QIAGEN), following the modified protocol [39]. For *H. feroniarum*, a 658-bp fragment of *cox1* was initially amplified by PCR with the primer pairs LCO1490–HCO2198 [40] (Additional file 7: Table S4). PCR products were purified and sequenced directly using the Sanger method at Majorbio (Shanghai, China). Specific primers for *H. feroniarum*, HCOIF1 and HCOIR1, were designed from the sequences of the *cox1* fragment. PCR with these two primers produced a 13.5-kb amplicon, which was sequenced with Illumina Hiseq 2000 platform at the Majorbio (Shanghai, China).

For *R. robini*, a 395-bp fragment of *cob* and a 357-bp fragment of *rrnS* were initially amplified by PCR with the primer pairs CytbF–CytbR [41] and SR-J-14199–SR-J-14199 [42] (see Additional file 7: Table S4). The PCR products were purified and sequenced directly using Sanger method at Majorbio. Two pairs of specific primers, R412SF1–R4COBR1 and R4COBF2–R412SR3, were designed from the sequences of the *cob* and *rrnS* fragments. The PCR with R412SF1–R4COBR1 produced a 5.8-kb amplicon. The PCR with R4COBF2–R412SR3 produced an 8.4-kb amplicon. Both amplicons were sequenced with Illumina Hiseq 2000 platform at the Majorbio.

The initial PCRs contained 12.5 μL of PCR SuperMix (Transgene Biotech Co., Ltd., Beijing, China), 2 μl of template DNA, and 1.25 μM of each primer, for a total volume of 25 μL. The PCR cycling conditions were: 3-min denaturation at 96 °C; 35 cycles of 10-s denaturation at 95 °C, 30-s annealing at 46 °C and 1.5-min extension at 72 °C; 5-min final extension at 72 °C. Then, the PCRs were held at 4 °C. PCR products were checked on 1% agarose gel. PrimeSTAR GXL DNA polymerase (TAKARA) was used in the long PCRs with the cycling conditions: 35 cycles of 98 °C for 10 s, 68 °C for 5 to 10 min (depends on the length of regions between *rrnS* and *cob*). The reaction mixture contained 0.5 μl GXL DNA Polymerase, 5 μl buffer, 2 μl dNTP mixture, 0.75 μl of each primer, 1 μl of template DNA and Milli-Q water added to a total volume of 25 μl. Positive and negative controls were executed with each PCR. PCR products were checked on 1% agarose gel. PCR products were purified with QIAquick Spin PCR Purification Kit (QIAGEN).

### Assembly of Illumina sequence-reads, gene identification and codon usage analysis

Illumina sequence-reads obtained from the mt genome amplicons of *H. feroniarum* and *R. robini* were assembled into contigs with Geneious 8.1.2 (Biomatters Ltd.). The tRNA genes were identified using tRNAscan-SE [26] and ARWEN [27] or identified manually based on anticodons and secondary structures. tRNA genes of the two sarcoptiform mites were verified by comparison of secondary structures and conserved nucleotide sequences with those of the Acari species reported in published literature. PCGs were identified by open reading frame search in Geneious and BLAST searches of GenBank [43]. The two rRNA genes, *rrnL* and *rrnS*, were also identified by BLAST searches of GenBank based on sequence similarity and conserved sequence motifs. The start and stop nucleotides of *rrnL* and *rrnS* cannot be determined exactly and were assumed to be immediately after their upstream genes and before their downstream genes. The nucleotide sequences of mt genomes of *H. feroniarum* and *R. robini* have been deposited in GenBank under accession numbers MF596167 and MF596168. The codon usage and Relative Synonymous Codon Usage (RSCU) values were analyzed with MEGA 6.0.6 [44].

### Retrieving the “lost” mt tRNA genes in sarcoptiform mites

Mitochondrial genome sequences of *T. longior*, *T. putrescentiae*, *Sa. scabiei* and *St. magnus* were retrieved from NCBI (Additional file 8: Table S5). We surveyed the “lost” tRNA genes using tRNAscan-SE [26] and ARWEN [27] and then manually identified the “lost” tRNA genes. We focused on the gap regions (46–215 bp) between identified genes where the anticodon sequences of the “lost” tRNA genes could be found. To find conserved nucleotides in anticodon loops, the nucleotide sequences of 17 “lost” tRNA genes (*trnA*, *trnC*, *trnD*, *trnE*, *trnF*, *trnG*, *trnI*, *trnK*, *trnL*_*1*_, *trnM*, *trnN*, *trnQ*, *trnR*, *trnS*_*1*_, *trnT*, *trnV* and *trnY*) of sarcoptiform mites were aligned using MUSCLE algorithm in Geneious 8.1.2. and manually formed the secondary structures. The nucleotide sequences of anticodon loops are relatively conserved in these sarcoptiform mites (Figs. 2, 3 and Additional file 4: Figure S2).

To get more species of oribatid mites for comparison with *Steganacarus magnus*, we also retrieved and analyzed the whole genome sequence data of three oribatid mites available in NCBI, i.e., *Platynothrus peltifer* (ID: 37201), *Hypochthonius rufulus* (ID: 37200), *Achipteria coleoptrata* (ID: 37199) (Additional file 8: Table S5). We searched the mt genome sequences and contigs of these oribatid mites from their whole genome sequence data using the sequence of their *cox1* as a reference. Unfortunately, the coverage of mt genomes is very low in the whole genome sequence data of these 3 oribatid mites. We found only mt *trnK* for *Pl. peltifer* from one contig (GenBank accession number LBFO01104924.1, Fig. 2) but no mt tRNA genes for *Hypochthonius rufulus* and *Achipteria coleoptrata*.

## Additional files

Additional file 1: **Figure S1.** Inferred secondary structure of 19 mitochondrial tRNA genes of *Histiostoma feroniarum* (Hf) and 22 mitochondrial tRNA genes of *Rhizoglyphus robini* (Rr). tRNA genes are labeled with the abbreviations of their corresponding amino acids. Dashes indicate Watson–Crick bonds; dots indicate bonds between U and G. Shared identical sequences between tRNA genes are circled in *H. feroniarum*. (PDF 3430 kb)

Additional file 2: **Table S1.** Mitochondrial genome organization of *Rhizoglyphus robini*. (DOCX 13 kb)

Additional file 3: **Table S2.** Mitochondrial genome organization of *Histiostoma feroniarum*. (DOCX 16 kb)

Additional file 4: **Figure S2.** The alignment of nucleotide sequences of nine mitochondrial putative tRNA genes (encoded by *trnA*, *trnE*, *trnF*, *trnI*, *trnQ*, *trnR*, *trnS*_*1*_, *trnV* and *trnY*) in the Sarcoptiformes mites. The conserved sequences in anticodon loops were marked. (PDF 4582 kb)

Additional file 5: **Table S3.** Amino acid frequencies of the sarcoptiform mites. (DOCX 16 kb)

Additional file 6: **Figure S3.** Relative synonymous codon usage (RSCU) and codon numbers for five amino acids (H, L_2_, P, S_2_ and W) in the mitochondrial genomes of sarcoptiform mites. The X-axis shows the sarcoptiform mites, and the Y-axis shows the RSCU or total number of codons. The blue column indicates the codons that match the anticodons of the corresponding mt tRNA genes. The red, green and purple column indicate the imperfect, synonymous codons to the anticodons of their corresponding mt tRNA genes. (PDF 635 kb)

Additional file 7: **Table S4.** PCR primers used in this study. (DOCX 13 kb)

Additional file 8: **Table S5.** Sarcoptiform mites included in this study. (DOCX 18 kb)

## Abbreviations

*atp6* and *atp8*: Genes for ATP synthase subunits 6 and 8
bp: Base pair
*cob*: Gene for cytochrome b
*cox1*, *cox2* and *cox3*: Genes for cytochrome c oxidase subunits 1, 2 and 3
CR: Control region
DNA: Deoxyribonucleic acid
kb: Kilo base pair
Mt.: Mitochondrial
*nad1*, *nad2*, *nad3*, *nad4*, *nad4L*, *nad5* and *nad6*: Mitochondrial genes for NADH dehydrogenase subunits 1–6 and 4 L
NCRs: Non-coding regions
PCGs: Protein-coding genes
PCR: Polymerase chain reaction
rRNA: Ribosomal RNA
*rrnS* and *rrnL*: Genes for small and large subunits of ribosomal RNA
RSCU: Relative Synonymous Codon Usage
*trnA* or A: tRNA gene for alanine
tRNA: Transfer RNA
*trnC* or C: tRNA gene for cysteine
*trnD* or D: tRNA gene for aspartic acid
*trnE* or E: tRNA gene for glutamic acid
*trnF* or F: tRNA gene for phenylalanine
*trnG* or G: tRNA gene for glycine
*trnH* or H: tRNA gene for histidine
*trnI* or I: tRNA gene for isoleucine
*trnK* or K: tRNA gene for lysine
*trnL*_*1*_ or L_1_: tRNA gene for leucine (anticodon NAG)
*trnL*_*2*_ or L_2_: tRNA gene for leucine (anticodon YAA)
*trnM* or M: tRNA gene for methionine
*trnN* or N: tRNA gene for asparagine
*trnP* or P: tRNA gene for proline
*trnQ* or Q: tRNA gene for glutamine
*trnR* or R: tRNA gene for arginine
*trnS*_*1*_ or S_1_: tRNA gene for serine (anticodon NCU)
*trnS*_*2*_ or S_2_: tRNA gene for serine (anticodon NGA)
*trnT* or T: tRNA gene for threonine
*trnV* or V: tRNA gene for valine
*trnW* or W: tRNA gene for tryptophan
*trnY* or Y: tRNA gene for tyrosine
μl: Microliter
