# Supplementary figures and images for: The mitochondrial genomes of sarcoptiform mites: are any transfer RNA genes really lost?
Source: BMC Genomics. 2018 Jun 18;19:466. doi: 10.1186/s12864-018-4868-6 (PMC6006854; doi:10.1186/s12864-018-4868-6)

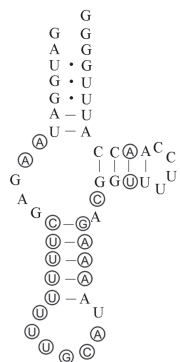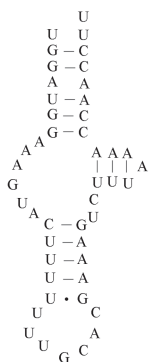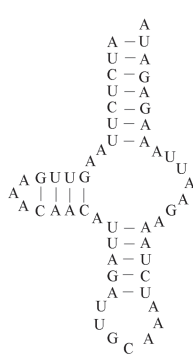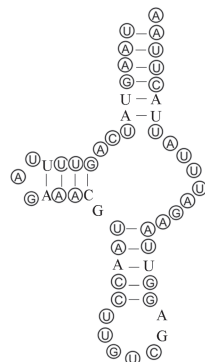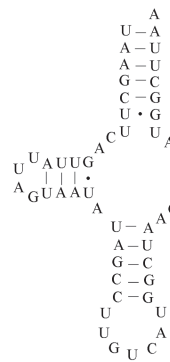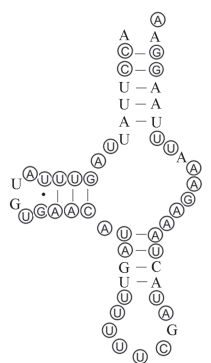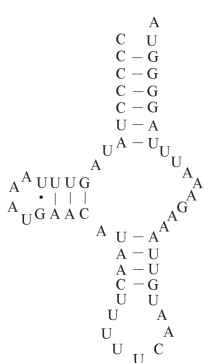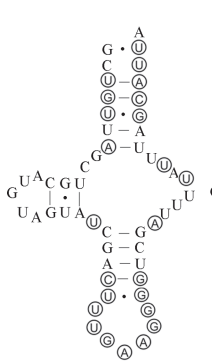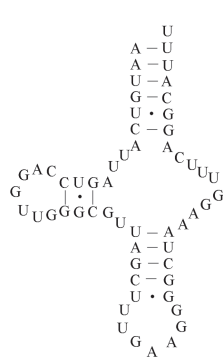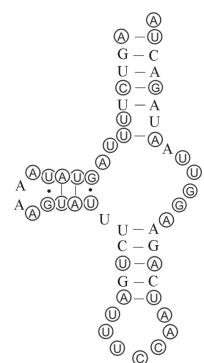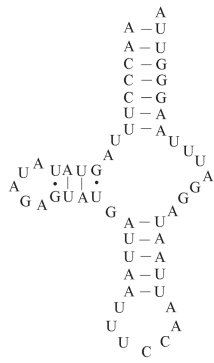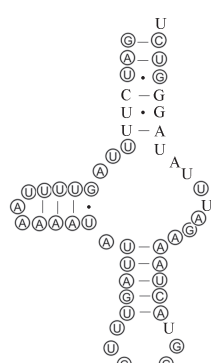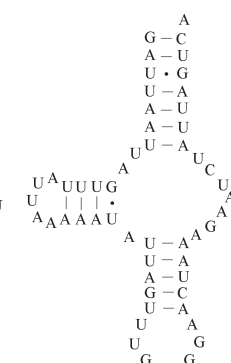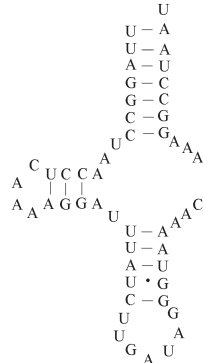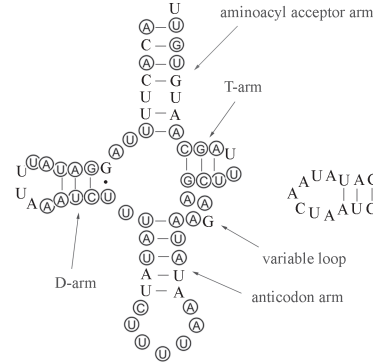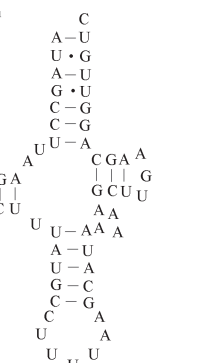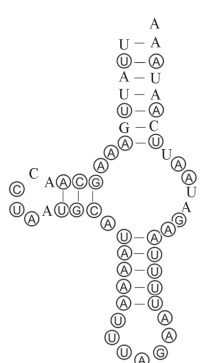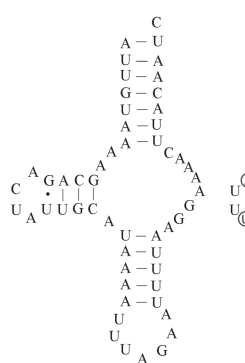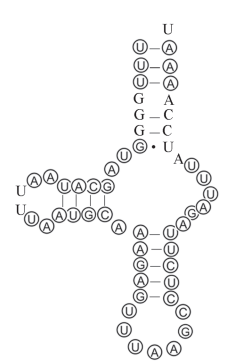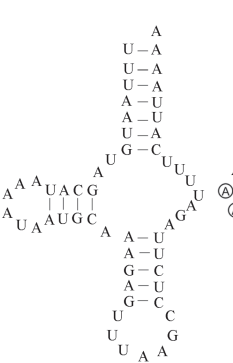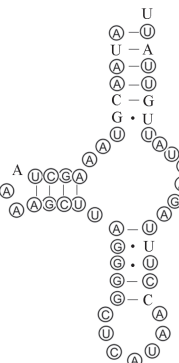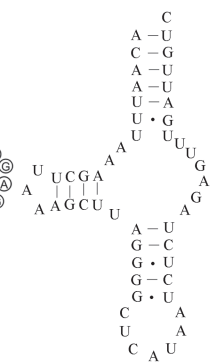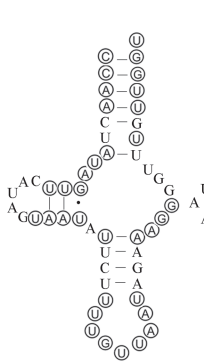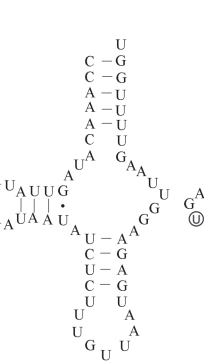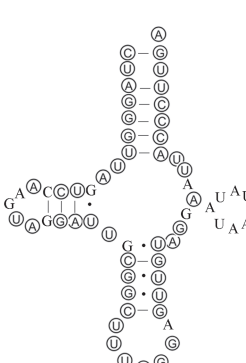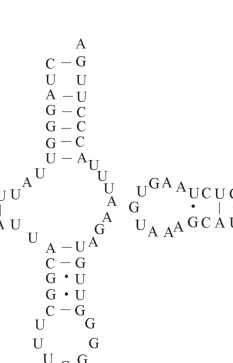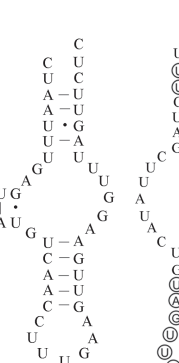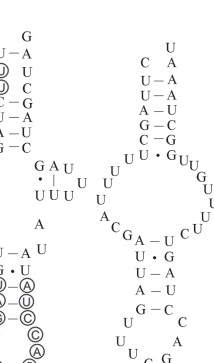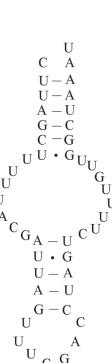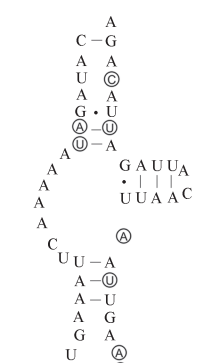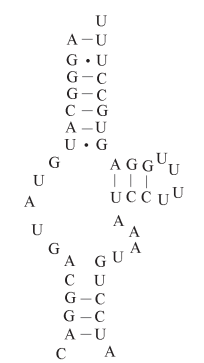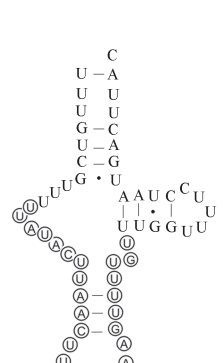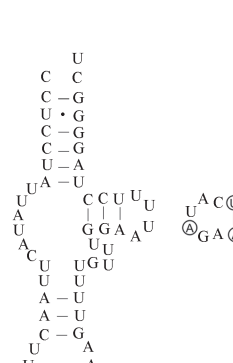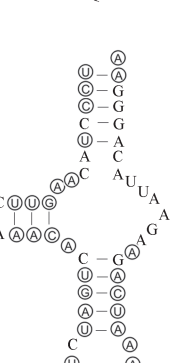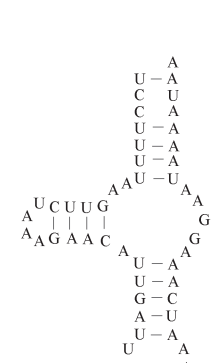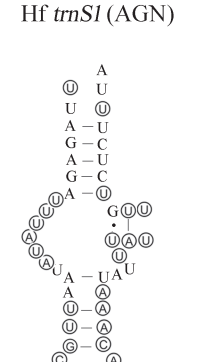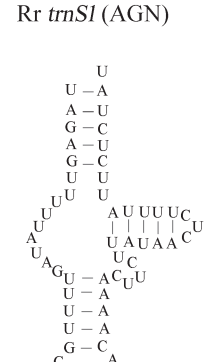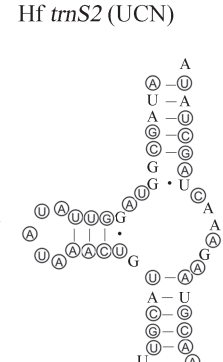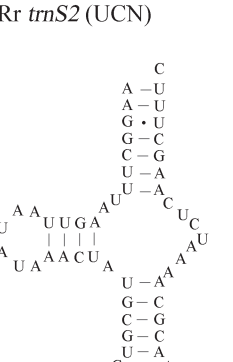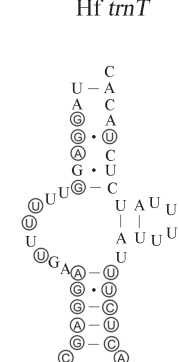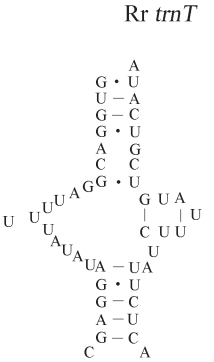

Supplement: Supplementary file 1 — Figure S1. Inferred secondary structure of 19 mitochondrial tRNA genes of Histiostoma feroniarum (Hf) and 22 mitochondrial tRNA genes of Rhizoglyphus robini (Rr). tRNA genes are labeled with the abbreviations of their corresponding amino acids. Dashes indicate Watson–Crick bonds; dots indicate bonds between U and G. Shared identical sequences between tRNA genes are circled in H. feroniarum. (PDF 3430 kb) [file 12864_2018_4868_MOESM1_ESM.pdf]

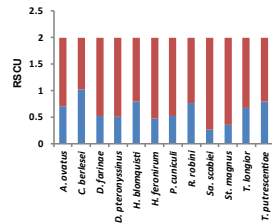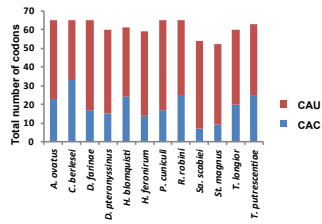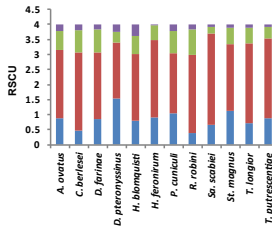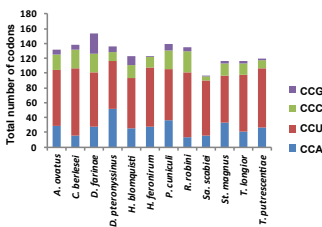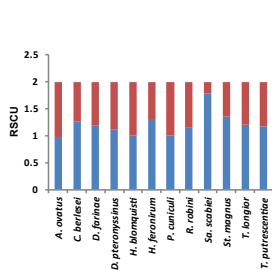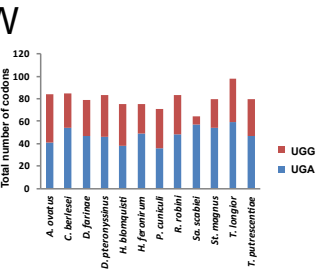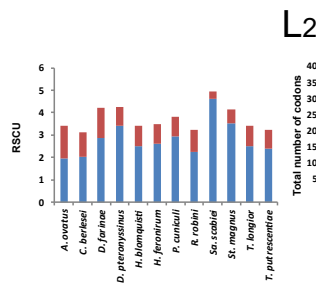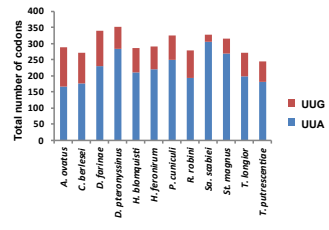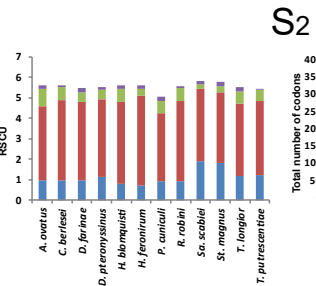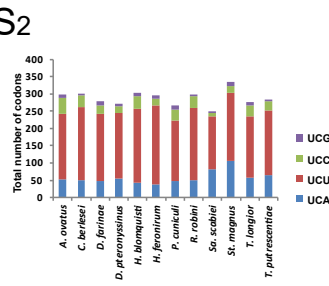

Supplement: Supplementary file 6 — Figure S3. Relative synonymous codon usage (RSCU) and codon numbers for five amino acids (H, L2, P, S2 and W) in the mitochondrial genomes of sarcoptiform mites. The X-axis shows the sarcoptiform mites, and the Y-axis shows the RSCU or total number of codons. The blue column indicates the codons that match the anticodons of the corresponding mt tRNA genes. The red, green and purple column indicate the imperfect, synonymous codons to the anticodons of their corresponding mt tRNA genes. (PDF 635 kb) [file 12864_2018_4868_MOESM6_ESM.pdf]
