# Supplementary material for: The mitochondrial genomes of sarcoptiform mites: are any transfer RNA genes really lost?
Source: BMC Genomics. 2018 Jun 18;19:466. doi: 10.1186/s12864-018-4868-6 (PMC6006854; doi:10.1186/s12864-018-4868-6)
Supplement: Supplementary file 2 — Table S1. Mitochondrial genome organization of Rhizoglyphus robini. (DOCX 13 kb) [file 12864_2018_4868_MOESM2_ESM.docx]

**Table S1** **Mitochondrial genome organization of *Rhizoglyphus robini***

| Gene | Strand | Position and intergenic nucleotides^a^ | Size | Start codon | Stop codon | Anti codon |
| --- | --- | --- | --- | --- | --- | --- |
| cox1 | J | 1–1536 (6) | 1536 | ATA | TAA |  |
| cox2 | J | 1543–2304 (2) | 762 | ATG | TAG |  |
| trnD | J | 2307–2361 (32) | 55 |  |  | GTC |
| atp8 | J | 2394–2516 (4) | 123 | ATA | TAG |  |
| atp6 | J | 2521–3192 (13) | 672 | ATG | TAG |  |
| cox3 | J | 3206–3988 (10) | 783 | ATG | TAA |  |
| trnG | J | 3999–4053 (-1) | 55 |  |  | TCC |
| nad3 | J | 4053–4397 (25) | 345 | ATT | TAG |  |
| trnR | J | 4423–4469 (15) | 47 |  |  | TCG |
| trnM | J | 4485–4536 (1) | 52 |  |  | CAT |
| trnS2 | J | 4538–4589 (2) | 52 |  |  | TGA |
| trnC | N | 4592–4644 (11) | 53 |  |  | GCA |
| trnP | J | 4656–4710 (2) | 55 |  |  | TGG |
| trnY | J | 4713–4763 (-3) | 51 |  |  | GTA |
| trnK | J | 4761–4823 (-1) | 63 |  |  | TTT |
| trnN | J | 4823–4877 (0) | 55 |  |  | GTT |
| rrnS | J | 4878–5538 (0) | 661 |  |  |  |
| trnV | J | 5539–5595 (0) | 57 |  |  | TAC |
| rrnL | J | 5596–6612 (0) | 1017 |  |  |  |
| trnW | J | 6613–6668 (53) | 56 |  |  | TCA |
| nad1 | N | 6722–7669 (33) | 948 | ATA | TAA |  |
| nad6 | N | 7703–8137 (3) | 435 | ATA | TAA |  |
| trnT | N | 8141–8192 (2) | 52 |  |  | TGT |
| nad4L | J | 8195–8446(12) | 252 | ATG | TAA |  |
| nad4 | J | 8459–9737 (0) | 1279 | ATG | T |  |
| trnH | J | 9738–9792 (-1) | 55 |  |  | GTG |
| nad5 | J | 9792–11421 (0) | 1630 | ATA | T |  |
| trnF | J | 11422–11479 (0) | 58 |  |  | GAA |
| CR |  | 11480–11798 (0) | 319 |  |  |  |
| trnS1 | N | 11799–11849 (0) | 51 |  |  | TCT |
| trnQ | N | 11850–11906 (-5) | 57 |  |  | TTG |
| trnI | N | 11902–11957 (-39) | 56 |  |  | GAT |
| nad2 | N | 11919–12902 (-1) | 984 | ATA | TAA |  |
| trnE | N | 12902–12956 (6) | 55 |  |  | TTC |
| cob | N | 12963–14063 (2) | 1001 | ATG | TAA |  |
| trnL1 | N | 14066–14120 (9) | 55 |  |  | TAG |
| trnA | J | 14130–14176 (-2) | 47 |  |  | TGC |
| trnL2 | J | 14175–14230 (14) | 56 |  |  | TAA |

^a^Negative numbers indicate overlapping nucleotides between adjacent genes
