# Supplementary material for: The mitochondrial genomes of sarcoptiform mites: are any transfer RNA genes really lost?
Source: BMC Genomics. 2018 Jun 18;19:466. doi: 10.1186/s12864-018-4868-6 (PMC6006854; doi:10.1186/s12864-018-4868-6)
Supplement: Supplementary file 3 — Table S2. Mitochondrial genome organization of Histiostoma feroniarum. (DOCX 16 kb) [file 12864_2018_4868_MOESM3_ESM.docx]

**Table S2 Mitochondrial genome organization of *Histiostoma feroniarum***

| Gene | Strand | Position and intergenic nucleotides^a^ | Size | Start codon | Stop codon | Anti codon |
| --- | --- | --- | --- | --- | --- | --- |
| cox1 | J | 1–1536 (6) | 1536 | ATA | TAG |  |
| cox2 | J | 1543–2310 (1) | 768 | ATA | TAA |  |
| trnD | J | 2312–2364 (-1) | 53 |  |  | GTC |
| atp8 | J | 2364–2519 (0) | 156 | ATT | TAA |  |
| atp6 | J | 2520–3188 (-1) | 669 | ATG | TAA |  |
| cox3 | J | 3188–3970 (-2) | 783 | ATG | TAG |  |
| trnG | J | 3969–4021 (-8) | 53 |  |  | TCC |
| nad3 | J | 4014–4364 (3) | 351 | ATA | TAA |  |
| trnR | J | 4368–4415 (0) | 48 |  |  | TCG |
| trnM | J | 4416–4467 (-1) | 52 |  |  | CAT |
| trnS2 | J | 4467–4522 (3) | 56 |  |  | TGA |
| trnV | J | 4526–4573 (4) | 48 |  |  | TAC |
| trnF | J | 4578–4631 (19) | 54 |  |  | GAA |
| trnP | J | 4651–4705 (4) | 55 |  |  | TGG |
| trnY | J | 4710–4759 (-4) | 50 |  |  | GTA |
| trnK | J | 4756–4816 (0) | 61 |  |  | TTT |
| trnN | J | 4817–4869 (0) | 53 |  |  | GTT |
| rrnS | J | 4870–5538 (0) | 669 |  |  |  |
| trnC | N | 5539–5587 (0) | 49 |  |  | ACA |
| rrnL | J | 5588–6584 (0) | 997 |  |  |  |
| CR1 |  | 6585–6684 (0) | 100 |  |  |  |
| trnI | N | 6685–6738 (-8) | 54 |  |  | AAT |
| nad1 | N | 6731–7645 (-7) | 915 | ATA | TAA |  |
| nad6 | N | 7639–8067 (-1) | 429 | ATT | TAA |  |
| trnT | N | 8067–8120 (1) | 54 |  |  | TGT |
| nad4L | J | 8122–8370 (0) | 249 | ATG | TAA |  |
| nad4 | J | 8371–9771 (0) | 1401 | ATG | TAG |  |
| CR2 |  | 9772–9914 (0) | 143 |  |  |  |
| trnH | J | 9915–9967 (-13) | 53 |  |  | GTG |
| nad5 | J | 9955–11586 (41) | 1632 | ATT | TAA |  |
| trnQ | J | 11628–11674 (-11) | 47 |  |  | CTG |
| trnW | J | 11664–11718 (45) | 55 |  |  | TCA |
| trnS1 | N | 11764–11813 (19) | 50 |  |  | TCT |
| nad2 | N | 11833–12576 (-1) | 744 | ATA | TAA |  |
| trnE | N | 12576–12629 (-1) | 54 |  |  | TTC |
| cob | N | 12629–13726 (-1) | 1098 | ATA | TAA |  |
| trnL1 | N | 13726–13779(4) | 54 |  |  | TAG |
| trnA | J | 13784–13834 (-7) | 51 |  |  | TGC |
| trnL2 | J | 13828–13883 (13) | 56 |  |  | TAA |

^a^Negative numbers indicate overlapping nucleotides between adjacent genes
