# Supplementary material for: The mitochondrial genomes of sarcoptiform mites: are any transfer RNA genes really lost?
Source: BMC Genomics. 2018 Jun 18;19:466. doi: 10.1186/s12864-018-4868-6 (PMC6006854; doi:10.1186/s12864-018-4868-6)
Supplement: Supplementary file 4 — Figure S2. The alignment of nucleotide sequences of nine mitochondrial putative tRNA genes (encoded by trnA, trnE, trnF, trnI, trnQ, trnR, trnS1, trnV and trnY) in the Sarcoptiformes mites. The conserved sequences in anticodon loops were marked. (PDF 4582 kb) [file 12864_2018_4868_MOESM4_ESM.pdf]

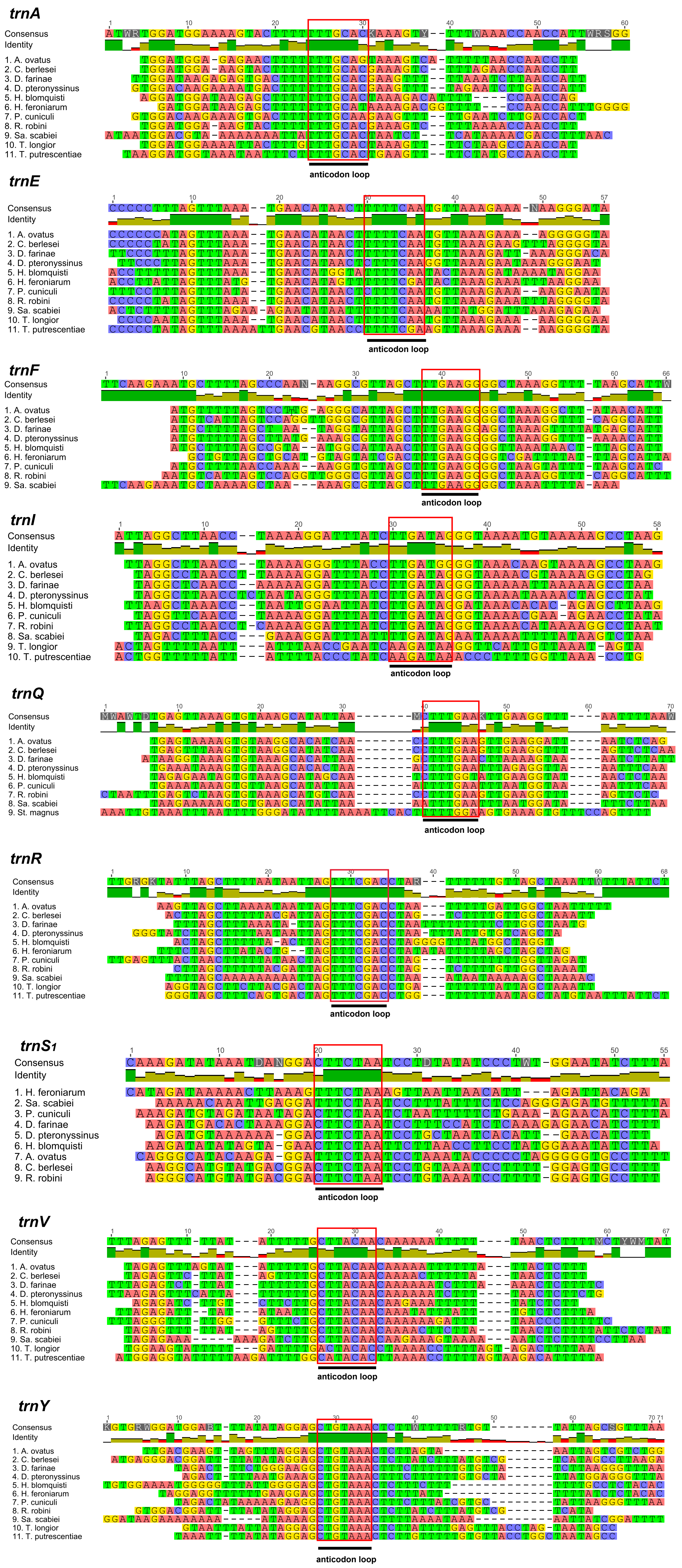

trnA
Consensus
Identity
1. A. ovatus
2. C. berlesei
3. D. farinae
4. D. pteronyssinus
5. H. blomquisti
6. H. feroniarum
7. P. cuniculi
8. R. robini
9. Sa. scabiei
10. T. longior
11. T. putrescentiae
anticodon loop
trnE
Consensus
Identity
1. A. ovatus
2. C. berlesei
3. D. farinae
4. D. pteronyssinus
5. H. blomquisti
6. H. feroniarum
7. P. cuniculi
8. R. robini
9. Sa. scabiei
10. T. longior
11. T. putrescentiae
anticodon loop
trnF
Consensus
Identity
1. A. ovatus
2. C. berlesei
3. D. farinae
4. D. pteronyssinus
5. H. blomquisti
6. H. feroniarum
7. P. cuniculi
8. R. robini
9. Sa. scabiei
anticodon loop
trnI
Consensus
Identity
1. A. ovatus
2. C. berlesei
3. D. farinae
4. D. pteronyssinus
5. H. blomquisti
6. P. cuniculi
7. R. robini
8. Sa. scabiei
9. T. longior
10. T. putrescentiae
anticodon loop
trnQ
Consensus
Identity
1. A. ovatus
2. C. berlesei
3. D. farinae
4. D. pteronyssinus
5. H. blomquisti
6. P. cuniculi
7. R. robini
8. Sa. scabiei
9. St. magnus
anticodon loop
trnR
Consensus
Identity
1. A. ovatus
2. C. berlesei
3. D. farinae
4. D. pteronyssinus
5. H. blomquisti
6. H. feroniarum
7. P. cuniculi
8. R. robini
9. Sa. scabiei
10. T. longior
11. T. putrescentiae
anticodon loop
trnS1
Consensus
Identity
1. H. feroniarum
2. Sa. scabiei
3. P. cuniculi
4. D. farinae
5. D. pteronyssinus
6. H. blomquisti
7. A. ovatus
8. C. berlesei
9. R. robini
anticodon loop
trnV
Consensus
Identity
1. A. ovatus
2. C. berlesei
3. D. farinae
4. D. pteronyssinus
5. H. blomquisti
6. H. feroniarum
7. P. cuniculi
8. R. robini
9. Sa. scabiei
10. T. longior
11. T. putrescentiae
anticodon loop
trnY
Consensus
Identity
1. A. ovatus
2. C. berlesei
3. D. farinae
4. D. pteronyssinus
5. H. blomquisti
6. H. feroniarum
7. P. cuniculi
8. R. robini
9. Sa. scabiei
10. T. longior
11. T. putrescentiae
anticodon loop
