# Supplementary material for: The mitochondrial genomes of sarcoptiform mites: are any transfer RNA genes really lost?
Source: BMC Genomics. 2018 Jun 18;19:466. doi: 10.1186/s12864-018-4868-6 (PMC6006854; doi:10.1186/s12864-018-4868-6)
Supplement: Supplementary file 5 — Table S3. Amino acid frequencies of the sarcoptiform mites. (DOCX 16 kb) [file 12864_2018_4868_MOESM5_ESM.docx]

**Table S3** Amino acid frequencies of the sarcoptiform mites

| Taxa | Amino acid (%) | | | | | | | | | | | | | | | | | | | | | |
| --- | --- | --- | --- | --- | --- | --- | --- | --- | --- | --- | --- | --- | --- | --- | --- | --- | --- | --- | --- | --- | --- | --- |
|  | A | C | D | E | F | G | H | I | K | L1 | L2 | M | N | P | Q | R | S1 | S2 | T | V | W | Y |
| *Aleuroglyphus ovatus* | 3.9 | 1.8 | 2.1 | 2.3 | 12.3 | 6.0 | 1.8 | 6.7 | 2.9 | 6.1 | 7.9 | 6.4 | 3.0 | 3.6 | 0.9 | 1.2 | 3.5 | 8.2 | 4.8 | 7.9 | 2.3 | 4.1 |
| *Caloglyphus berlesei* | 3.8 | 1.7 | 2.1 | 2.4 | 11.2 | 6.2 | 1.8 | 6.1 | 3.0 | 7.0 | 7.5 | 6.3 | 2.7 | 3.8 | 1.0 | 1.2 | 3.6 | 8.3 | 4.6 | 9.0 | 2.4 | 4.3 |
| *Dermatophagoides farinae* | 2.8 | 1.9 | 1.9 | 2.4 | 12.7 | 5.7 | 1.8 | 7.3 | 3.5 | 4.0 | 9.4 | 7.3 | 3.3 | 4.3 | 1.1 | 1.1 | 3.5 | 7.7 | 4.2 | 8.2 | 2.2 | 4.2 |
| *Dermatophagoides pteronyssinus* | 2.8 | 1.7 | 2.0 | 2.3 | 12.0 | 5.8 | 1.7 | 8.1 | 3.4 | 4.1 | 9.8 | 7.3 | 3.4 | 3.8 | 1.0 | 1.1 | 3.4 | 7.6 | 4.1 | 7.8 | 2.3 | 4.6 |
| *Histiostoma blomquisti* | 3.7 | 1.3 | 2.0 | 2.0 | 12.5 | 5.7 | 1.7 | 7.5 | 2.7 | 6.1 | 8.0 | 8.2 | 2.6 | 3.4 | 1.1 | 1.1 | 3.6 | 8.4 | 4.7 | 7.4 | 2.1 | 3.9 |
| *Histiostoma feronirum* | 2.9 | 1.7 | 2.0 | 2.2 | 15.3 | 5.2 | 1.7 | 7.0 | 2.9 | 5.9 | 8.1 | 7.3 | 2.9 | 3.5 | 1.1 | 1.0 | 3.5 | 8.3 | 4.3 | 7.2 | 2.1 | 3.8 |
| *Psoroptes cuniculi* | 3.0 | 2.1 | 1.9 | 2.1 | 12.3 | 5.3 | 1.8 | 7.2 | 3.5 | 5.2 | 9.0 | 7.3 | 3.6 | 3.9 | 1.1 | 1.1 | 4.3 | 7.4 | 3.8 | 7.8 | 2.0 | 4.2 |
| *Rhizoglyphus robini* | 4.0 | 1.7 | 2.1 | 2.4 | 11.5 | 6.2 | 1.8 | 6.2 | 3.0 | 6.6 | 7.7 | 6.3 | 2.8 | 3.7 | 1.0 | 1.1 | 3.6 | 8.3 | 4.5 | 8.8 | 2.3 | 4.3 |
| *Sarcoptes scabiei* | 2.2 | 1.0 | 1.7 | 2.2 | 13.2 | 4.3 | 1.6 | 9.4 | 5.3 | 2.1 | 10.0 | 10.0 | 5.3 | 3.0 | 1.2 | 1.0 | 2.9 | 7.6 | 4.5 | 4.4 | 1.9 | 5.3 |
| *Steganacarus magnus* | 2.4 | 1.0 | 1.9 | 2.8 | 12.5 | 5.0 | 1.5 | 10.6 | 3.7 | 4.0 | 9.0 | 7.0 | 4.1 | 3.3 | 1.4 | 1.2 | 3.6 | 9.5 | 3.5 | 6.0 | 2.3 | 3.6 |
| *Tyrophagus longior* | 3.4 | 1.9 | 2.3 | 2.1 | 12.1 | 5.4 | 1.7 | 6.5 | 3.4 | 5.9 | 7.8 | 6.5 | 3.2 | 3.3 | 1.3 | 1.4 | 3.6 | 7.9 | 5.1 | 7.7 | 2.8 | 4.6 |
| *Tyrophagus putrescentiae* | 3.6 | 1.5 | 2.1 | 2.3 | 11.4 | 5.4 | 1.8 | 7.4 | 3.2 | 6.1 | 7.1 | 6.8 | 3.1 | 3.5 | 1.1 | 1.2 | 3.8 | 8.2 | 5.2 | 8.2 | 2.3 | 4.7 |
