# Supplementary material for: The mitochondrial genomes of sarcoptiform mites: are any transfer RNA genes really lost?
Source: BMC Genomics. 2018 Jun 18;19:466. doi: 10.1186/s12864-018-4868-6 (PMC6006854; doi:10.1186/s12864-018-4868-6)
Supplement: Supplementary file 7 — Table S4. PCR primers used in this study. (DOCX 13 kb) [file 12864_2018_4868_MOESM7_ESM.docx]

**Table S4** PCR primers used in this study.

| Primer | Sequence (5’-3’) | Source |
| --- | --- | --- |
| LCO1490 | GGTCAACAAATCATAAAGATATTGG | Folmer et al. (1994) |
| HCO2198 | TAAACTTCAGGGTGACCAAAAAATCA | Folmer et al. (1994) |
| CytbF | GGWTAYGTWYTWCCWTGRGGWCARAT | Boore & Brown (2000) |
| CytbR | GCRTAWGCRAAWARRAARTAYCAYTCWGG | Boore & Brown (2000) |
| SR-J-14199 | TACTATGTTACGACTTAT | Kambhampati & Smith (1995) |
| SR-N-14594 | AAACTAGGATTAGATACCC | Kambhampati & Smith (1995) |
| HCOIF1 | GTTGGTACTGGTTGAACTGTTTATCCTCCTCTGTC | This study |
| HCOIR1 | CAGGTTGGCTTAACTCCAAACGAATAAGAACTCTA | This study |
| R412SF1 | GCGGGTTATCAATTATACTTAACAAGCTCCTCTGT | This study |
| R412SR3 | TGGTGTAAGTACATATCGCCCGTCACTCTTTTG | This study |
| R4COBF2 | TTTCCCTTCACTTTCTAATTCCTCTGGTTGTTG | This study |
| R4COBR1 | AGGCAGCAACAACCAGAGGAATTAGAAAGTGAAGG | This study |
