# Supplementary material for: The mitochondrial genomes of sarcoptiform mites: are any transfer RNA genes really lost?
Source: BMC Genomics. 2018 Jun 18;19:466. doi: 10.1186/s12864-018-4868-6 (PMC6006854; doi:10.1186/s12864-018-4868-6)
Supplement: Supplementary file 8 — Table S5. Sarcoptiform mites included in this study. (DOCX 18 kb) [file 12864_2018_4868_MOESM8_ESM.docx]

**Table S5.** Sarcoptiform mites included in this study.

| Supercohort | Cohort | Superfamily | Family | Species | GenBank number | Size (bp) | Reference |
| --- | --- | --- | --- | --- | --- | --- | --- |
| Enarthronotides |  | Hypochthonoidea | Hypochthoniidae | *Hypochthonius rufulus* | LBFL00000000.1 |  | Direct submission |
| Mixonomatides |  | Phthiracaroidea | Steganacaridae | *Steganacarus magnus* | EU935607 | 13,818 | Domes *et al*. 2008 |
| Desmonomatides | Nothrina | Crotonioidea | Camisiidae | *Platynothrus peltifer* | LBFO00000000.1 |  | Direct submission |
|  | Brachypylina | Achipterioidea | Achipteriidae | *Achipteria coleoptrata* | LBFM00000000.1 |  | Direct submission |
|  | Astigmatina | Acaroidea | Acaridae | *Aleuroglyphus ovatus* | KC700022 | 14,328 | Sun *et al*. 2014 |
|  |  |  |  | *Caloglyphus berlesei* | KF499016 | 14,273 | Sun *et al*. 2014 |
|  |  |  |  | *Rhizoglyphus robini* | MF596168 | 14,244 | This study |
|  |  |  |  | *Tyrophagus longior* | KR869095 | 13,271 | Yang & Li 2016 |
|  |  |  |  | *Tyrophagus putrescentiae* | KJ598129 | 13,288 | Que *et al*. 2014 |
|  |  | Analgoidea | Pyroglyphidae | *Dermatophagoides pteronyssinus* | EU884425 | 14,203 | Dermauw *et al*. 2009 |
|  |  |  |  | *Dermatophagoides farinae* | NC_013184 | 14,266 | Klimov & OConnor 2009 |
|  |  | Histiostomatoidea | Histiostomatidae | *Histiostoma blomquisti* | KX452726 | 15,892 | Lee & Wang, 2016 |
|  |  |  |  | *Histiostoma feronirum* | MF596167 | 13,896 | This study |
|  |  | Sarcoptoidea | Psoroptidae | *Psoroptes cuniculi* | KJ957822 | 14,247 | Gu *et al*. 2014 |
|  |  |  | Sarcoptidae | *Sarcoptes scabiei* | CM003133 | 13,667 | Mofiz *et al*. 2016 |
